# Assessment of the Cobas Bio centrifugal analyser

**DOI:** 10.1155/S1463924682000078

**Published:** 1982

**Authors:** Renze Bais, Robyn G. White, Donald Elston, Terence D. Geary

**Affiliations:** Division of Clinical Chemistry Institute of Medical and Veterinary Science Box 14 Rundle St. P.O. Adelaide South Australia 5000 Australia


Journal of Automatic Chemistry, Volume 4, Number 1 (January–March 1982), pages 21–24

### Assessment of the Cobas Bio centrifugal analyser

**Renze Bais, Robyn G. White, Donald Elston and Terence D. Geary**

*Division of Clinical Chemistry, Institute of Medical and Veterinary Science, Box 14, Rundle St. P.O., Adelaide, South Australia 5000, Australia*

### The instrument

The Cobas Bio (Roche) is a discrete spectrometric centrifugal analyser which is capable of monitoring 29 reaction cuvettes. The dispensing and analysing systems are contained within one unit and an Intel 8080A microprocessor controls the total system.

Two Hamilton syringes are used to pipette samples and reagents. The sample and reagent syringes have nominal capacities of 100 and 400 μl respectively. A second reagent can be pipetted by the sample syringe at a time interval of between 10 and 999 s.

Up to 25 sample cups are housed in coded discs which form the basis of an identification system. The sample cups are capped to minimize evaporation. They can be placed in one of two positions and the probe will only sample, by piercing the cup, those cups which are in the down position. This provides a way of selectively running different methods on the same sample plate.

The reagent boat contains receptacles for the primary and secondary reagents and up to three standards. The reagent pipettor uses disposable plastic tips, whereas the stainless-steel tip of the sample pipettor is automatically rinsed between samples.

The disposable rotor constructed from acrylic butadiene styrene polymer has 30 cuvettes, one of which is used to optimize the photometer light-source characteristics. A program allows for the effective use of empty cuvettes and the rotor need only be removed when all the cuvettes have been used. If the photometer arm is raised or the instrument turned off, this program is reset and the rotor must be replaced.

The detector unit consists of a digitally-controlled concave halographic grating monochromator, a central processing unit controlled energy adjustment system and a photodiode detector. The pulsed Xenon flash-tube has a spectral range of 285–750 nm.

Unlike other centrifugal analysers, the optical path-length is not fixed as the Cobas Bio measures absorbance in the same direction as the centrifugal force (see figure 1). The calculation factor is therefore dependent only on the selected sample volume and not the ratio of sample to reagent volume (Eisenwiener and Keller [1]). This allows the addition of a second reagent without altering the calculation factor (Eisenwiener *et al.* [2]).

Space is available for 30 programs, 10 have a key specifically allocated and the remainder are selected via a code number. Input is effected through the keyboard and there is considerable flexibility in the selection of program parameters. After an analytical run the data can be manipulated and a modified print-out obtained.

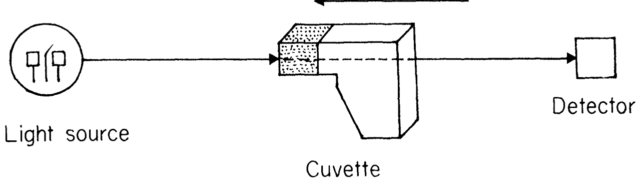

*Figure 1. Schematic diagram illustrating the measurement of absorbance in the same direction as the centrifugal force.*

### Evaluation

### *The dispensing system*

Since the results are only dependent on the selected sample volume when the reagent does not absorb, the precision and accuracy of the sample pipettor is of primary importance to the function of the Cobas Bio.

#### *Sample pipettor imprecision*

Sodium nitrite solution, which absorbs at 340 nm, was pipetted into each of 20 cuvettes and the absorbances measured. The imprecision was assessed at different sample volumes and gave coefficients of variation between 1·06% at 5 μl and 0·48% at 80 μl indicating that the intra-batch variation is acceptable.

#### *Sample pipettor inaccuracy*

A rotor was weighed before and after the addition of 80 μl of water to all cuvettes. The results showed a mean error of 0·066% per cuvette.

A plot of absorbance versus path-length was constructed from various volumes of sodium nitrite pipetted into the rotor. The results indicated that absorbance is linearly related to path-length (figure 2). The accuracy of the pipetting system was

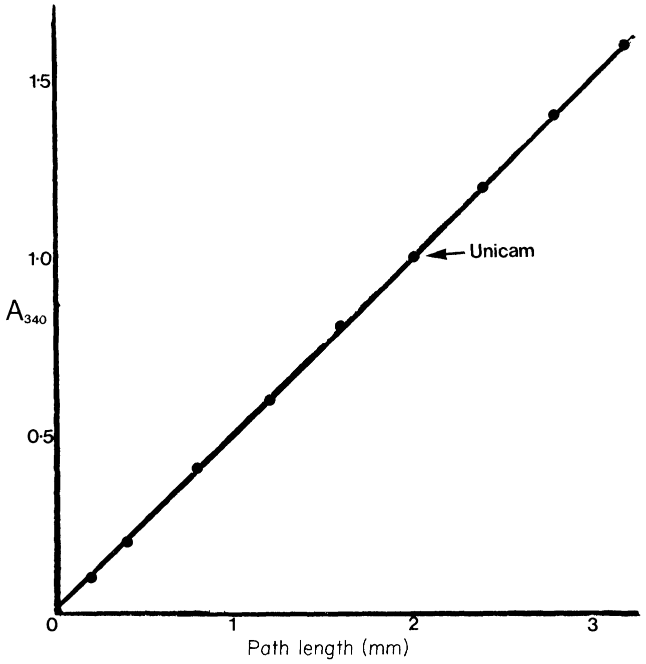

*Figure 2. Linearity of absorbance of sodium nitrite versus path-length indicating the linear relationship between absorbance and volume pipetted into the rotor.*

0142-0453/82/0401 0021 $05·00 © 1982 Taylor & Francis Ltd confirmed by comparing the absorbances obtained on the Cobas Bio with those obtained on two spectrometers which had been previously standardized with solutions from the National Bureau of Standards (USA).

*Carry-over*

The only possible source of carry-over is from the sample pipettor. Using high and low levels of triglyceride and Young and Gochmann's method [3] it was demonstrated that the carry-over between samples was negligible (0·31%).

### *Analytical system precision*

*Photometer noise*

Various concentrations of sodium nitrite were pipetted in duplicate into a rotor by the sample pipettor and 21 readings taken at 30 s intervals. The results indicated that the noise level at an absorbance of 3·3 was acceptable but at 4·1 absorbance units there was a significant increase in the noise level.

*Rotor variation*

Five rotors were filled with sodium nitrite solution and the absorbances measured at 340 nm. For a mean absorbance of 0·83 absorbance units, the coefficient of variation for the five rotors ranged from 0·36% to 0·65%. As the difference in absorbance readings is a measure of the variation between cuvettes and the electronic noise of the instrument, this indicates that there is no significant difference between cuvettes of each rotor or between cuvettes from rotor to rotor.

*Wavelength inaccuracy*

The accuracy was confirmed by measuring, at various wavelengths, sodium nitrite and methylene blue solutions pipetted in duplicate. The absorbances obtained were compared to the results obtained with a Varian 634 recording spectrometer (Varian Techtron Pty. Ltd, Springvale, Victoria, Australia) standardized by using holmium filters and a mercury lamp.

*Photometer linearity*

Dilutions of the stock solutions shown in table 1 were pipetted in duplicate into a rotor and the absorbances measured at the appropriate wavelength. The photometer was linear to an absorbance of at least 2·0 for all wavelengths tested except at 660 nm where it was linear to 1·5 absorbance units.

*Table 1. Stock solutions used for assessing photometer linearity.*

| Wavelength (nm) | Solution |
|---|---|
| 285 | 0·5 g tryptophan in 50 ml 0·1 N NaOH |
| 340 | 5·0 g sodium nitrite in 100 ml water |
| 500 | Washed red blood-cells haemolysed with an equal volume of detergent |
| 540 | As for 500 nm |
| 575 | As for 500 nm |
| 660 | 0·1 g methylene blue in 100 ml water |

*Temperature control*

The absorbance of 3,5 dinitro-salicylic acid (DNS) varies linearly with temperatures between 20 and 40°C (Oliver and Scott [4]). A 100 mmol/l solution of DNS in 0·08 N potassium hydroxide (KOH) was prepared and analysed at 520 nm. The results confirmed that the plot of absorbance against temperature was linear and it was possible to show that the temperature settings were accurate by comparing the absorbance obtained at various temperatures on the Varian 634 recording spectrometer.

It was noticed that the DNS in the cuvettes took up to 2 min. longer to equilibrate to the selected temperature than indicated by the instrument. This delay may be significant in assays where there is a short lag-time before the first readings are taken.

### Total system performance

The total system performance was tested by measuring glucose, urate, triglyceride and asparate aminotransferase (AST), with methods recommended by the manufacturer. In addition, the start method was used for triglyceride and AST. This entailed the addition of a secondary reagent-glycerol kinase for triglyceride and α-ketoglutarate for AST—after pre-incubation of the serum with the primary reagent.

### *Imprecision*

(1) *Inter-batch*

Three controls were assayed in each of 20 batches and Tonks's criteria [5] were applied to assess the acceptability i.e. 2 × coefficient of variation % < allowable limits of error (ALE). The imprecision for all methods investigated was acceptable (table 2).

(2) *Intra-batch*

Three controls were each assayed 20 times in a single run and the coefficient of variation calculated (table 2). The intra-batch imprecision was less than the inter-batch imprecision at the same concentration. If the same criteria of acceptability are applied then the intra-batch imprecision for all analytes is acceptable.

*Table 2. Method imprecision.*

*Table 2(a): Inter-batch imprecision.*

*Control A*

| Analyte | Mean | S.D. | 2 C.V.% | ALE |
|---|---|---|---|---|
| Glucose | 2·47 (mmol/l) | 0·07 | 5·26 | 10·0 |
| Urate | 0·334 (mmol/l) | 0·01 | 7·12 | 10·0 |
| Triglyceride | 0·95 (mmol/l) | 0·03 | 5·62 | 10·0 |
| AST | 23·9 (U/l) | 0·99 | 8·28 | 20·0 |

*Control B*

| Analyte | Mean | S.D. | 2 C.V.% | ALE |
|---|---|---|---|---|
| Glucose | 5·25 (mmol/l) | 0·16 | 6·14 | 10·0 |
| Urate | 0·461 (mmol/l) | 0·01 | 3·62 | 10·0 |
| Triglyceride | 1·79 (mmol/l) | 0·05 | 5·40 | 10·0 |
| AST | 80·1 (U/l) | 3·59 | 8·97 | 20·0 |

*Control C*

| Analyte | Mean | S.D. | 2 C.V.% | ALE |
|---|---|---|---|---|
| Glucose | 12·23 (mmol/l) | 0·38 | 6·13 | 10·0 |
| Urate | 0·576 (mmol/l) | 0·009 | 3·08 | 10·0 |
| Triglyceride | 3·87 (mmol/l) | 0·07 | 3·60 | 10·0 |
| AST | 262·4 (U/l) | 2·44 | 1·86 | 20·0 |

*Table 2(b). Intra-batch imprecision.*

*Control A*

| Analyte | Mean | S.D. | C.V.% |
|---|---|---|---|
| Glucose | 2·46 (mmol/l) | 0·03 | 1·2 |
| Urate | 0·324 (mmol/l) | 0·004 | 1·10 |
| Triglyceride | 0·93 (mmol/l) | 0·01 | 1·28 |
| AST | 22·6 (U/l) | 0·8 | 3·24 |

*Control B*

| Analyte | Mean | S.D. | C.V.% |
|---|---|---|---|
| Glucose | 5·34 (mmol/l) | 0·04 | 0·81 |
| Urate | 0·474 (mmol/l) | 0·004 | 0·89 |
| Triglyceride | 1·75 (mmol/l) | 0·05 | 2·94 |
| AST | 80·9 (U/l) | 1·0 | 1·26 |

*Control C*

| Analyte | Mean | S.D. | C.V.% |
|---|---|---|---|
| Glucose | 12·11 (mmol/l) | 0·13 | 1·06 |
| Urate | 0·579 (mmol/l) | 0·007 | 1·16 |
| Triglyceride | 3·90 (mmol/l) | 0·04 | 1·03 |
| AST | 285·1 (U/l) | 1·53 | 0·59 |

### *Patient comparison*

Eighty patient samples were assayed in duplicate by the test method and the results compared with those obtained by the routine method of analysis in this laboratory. The principle of each method is summarized in table 3.

The test method was considered unacceptable if:

(1) The bias exceeds the allowable limits of error.
(2) The number of false clinical decisions exceeds 5%.

Figure 3 shows the satisfactory agreement between the test methods determined on the Cobas Bio and the comparative methods determined on either a Technicon SMAC or an Abbott ABA 100. There was no significant difference between the normal and start methods for either triglyceride or AST. The deviation of the regression line from $X = Y$ is explained by the difference between methods used.

*Table 3. Method principles.*

| Analyte | Cobas Bio | Routine |
|---|---|---|
| Glucose | Hexokinase | Glucose oxidase |
| Urate | Uricase | Alkaline phosphotungstate |
| Triglyceride | Lipase/glycerokinase | Lipase/glycerokinase |
| AST | NADH→NAD | NADH→NAD |

### *Practicability*

(1) *Cost of consumables*

If every cuvette in the rotor is used, and all items are used once only, the cost of consumables is low. Total cost will increase if some of the cuvettes are not used, but it can be reduced by washing the consumables and by assembling a full rotor from the unused sections.

(2) *Flexibility*

The analyser is simple to program for new methods and because of this the chemistries are not restricted to those supplied by Roche.

(3) *Throughput*

The Cobas Bio is an efficient discrete analyser in that a full rotor can be analysed in 3·5 min. plus the assay time. However, unlike a number of centrifugal analysers which have separate loader and analyser modules, the next rotor cannot be loaded until the analytical run is completed.

(4) *'Creeping'*

In a number of centrifugal analysers creeping of detergent-containing reagents between compartments in the cuvette causes premature mixing of the sample and reagents. The Cobas Bio rotor's design prevents this mixing.

### Summary of technical evaluation

*Compliance with Standards Association of Australia—Australian Standard C100*

Checks were made on electrical safety, noise immunity, and immunity to electrical supply fluctuations (±10% of 240V), which were all found to be acceptable.

The Cobas Bio complied with all clauses of the ASC100 investigated, apart from the following:

*Clause 3.2:* materials and equipment to be suitable for conditions of use.

(1) Wiring in the area of the direct current (DC) power-supplies showed considerable abrasion of electrical insulation due to the rough nature of the outer fibre-glass cover.
(2) Internal wiring in contact with waste-pump armature and armature vibration may cause damage.
(3) The system is not totally protected from spillages.

*Clause 3.5:* workmanship—the rework on a number of the printed circuit boards was below standard with flux left on boards and suspect solder-joints.

*Clause 5.4.7:* arrangement of equipment wiring—mains terminations were not suitably restrained to prevent contact with external metal parts.

### Discussion

The Cobas Bio is a compact unit and occupies a small area of laboratory-bench space when compared to other centrifugal analysers currently available in Australia. Desirable features of the instrument are that samples can be loaded without having to transfer the rotor from the loader to the analyser, and the positive identification of samples means that specimens can be discretely sampled for individual tests. The rotors are disposable thus eliminating the washing-cycle and errors caused by unsatisfactory cleaning procedures.

The few inadequacies of the Cobas Bio include first that the loader module can only hold 25 specimens, whereas the rotor has cuvettes for 29 samples. This means the operator must be present to change sample discs when a full rotor is to be run. Secondly, the high-pitched noise from the DC motor during analysis initially irritated staff not working on the system. Thirdly, the system takes up to 2 min. longer to equilitrate to the selected temperature than is indicated by the display.

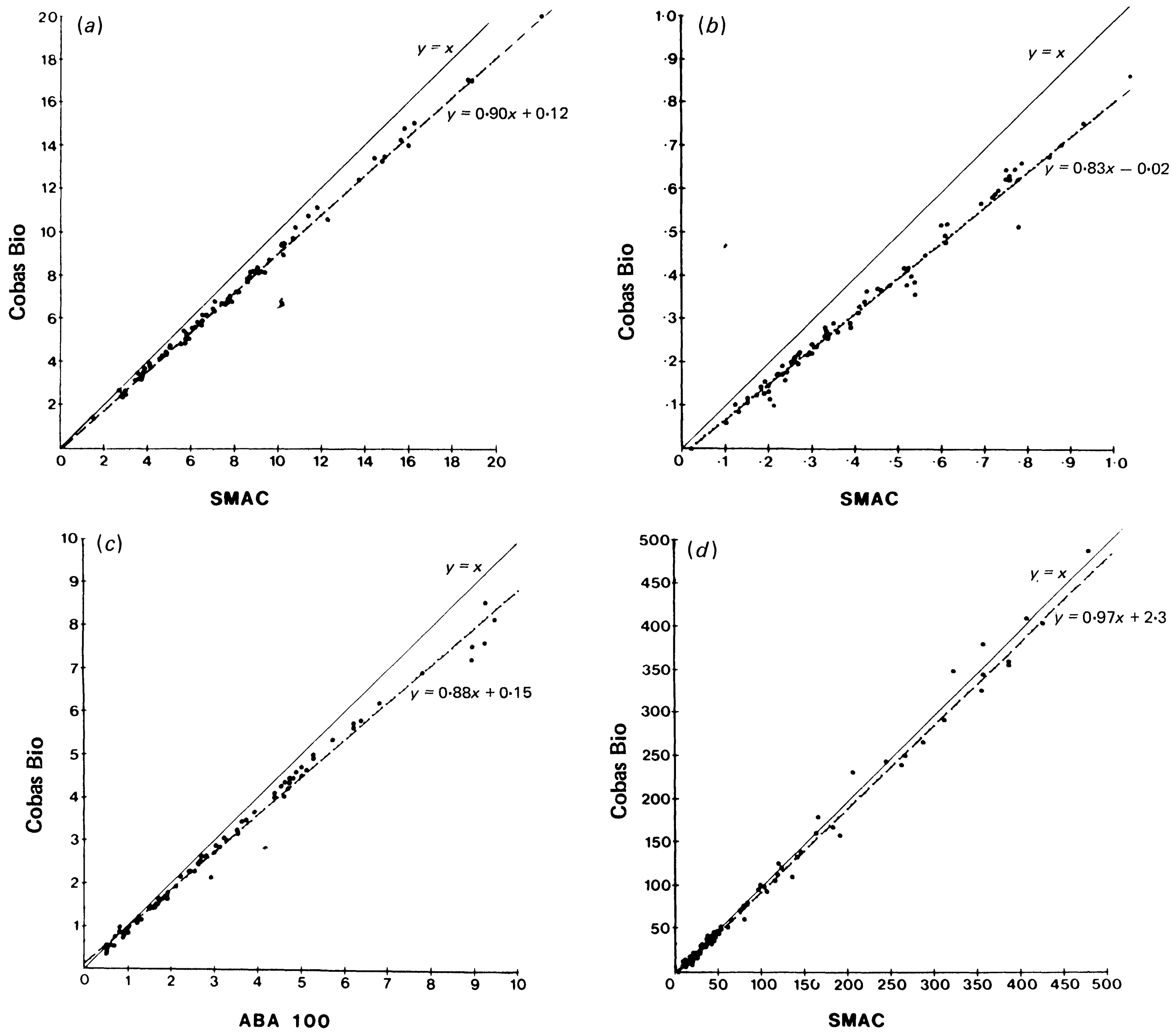

*Figure 3. Results of patient comparison between the Cobas Bio and routine laboratory methods. Figure 3(a) represents glucose (mmol/l); (b) urate (mmol/l); (c) triglyceride (mmol/l); and (d) asparate aminotransferase (U/l).*

The imprecision and inaccuracy of the Cobas Bio are well within acceptable limits, and the detector module was shown to be comparable in many respects to the reference-recording spectrometer.

Programming is simple to perform and, for routine use, the fact that once a run has commenced the parameters are locked preventing accidental alteration of the values, is a desirable feature. The single-button test-selection saves operator time and there is adequate throughput in both start and batch analysis mode.

A number of features of the instrument make it suitable for research and development work including the ease of programming, the ability to set wavelengths in 1 nm increments, addition of a second reagent and measurement of absorbance parallel to the cuvette. However, the flexibility could be increased with a few simple modifications. For research and development it would be desirable for the operator to be able to alter the parameters during analysis if he wishes. In start mode, only one reading is taken before the addition of the second reagent. If more readings could be taken then blank reactions could be measured. In addition, the rotor cannot be removed and replaced during an analytical run. This limits the capacity of the instrument in experiments where a long incubation time is required.

The Cobas Bio performed well during the evaluation period and staff quickly became familiar with its operation and enjoyed its simplicity. If the above modifications were made the flexibility of the system would be considerably increased, making it more suited for research procedures.

### References

1. Eisenwiener, H. G. and Keller, M., *Clinical Chemistry*, **25** (1979), 117–121.
2. Eisenwiener, H. G., Kindbeiter, J. M., Keller, M. and Gutlin, K., in *Centrifugal Analysers in Clinical Chemistry*, Ed. Price, C. P. and Spencer, K. (Praeger Publishers, 1980), 29–50.
3. Young, D. S. and Gochman, N., *Standard Methods of Clinical Chemistry*, 7 (1972), 293.
4. Oliver, R. W. A. and Scott, A., *Journal of Physics*, **7** (1974), 280.
5. Tonks, D. B., *Canadian Journal of Medical Technology*, **30** (1978), 38.

